# Differential Regulation of the Asthmatic Phenotype by the Aryl Hydrocarbon Receptor

**DOI:** 10.3389/fphys.2021.720196

**Published:** 2021-10-21

**Authors:** Hussein Traboulsi, Angela Rico de Souza, Benoit Allard, Zahraa Haidar, Mark Sorin, Vanessa Moarbes, Elizabeth D. Fixman, James G. Martin, David H. Eidelman, Carolyn J. Baglole

**Affiliations:** ^1^Meakins-Christie Laboratories, Research Institute of the McGill University Health Centre, Montreal, QC, Canada; ^2^Translational Research in Respiratory Diseases Program at the Research Institute of the McGill University Health Centre, Montreal, QC, Canada; ^3^Department of Medicine, McGill University, Montreal, QC, Canada; ^4^Department of Pathology, McGill University, Montreal, QC, Canada; ^5^Department of Pharmacology and Therapeutics, McGill University, Montreal, QC, Canada

**Keywords:** aryl hydrocarbon receptor, allergic asthma, chlorine, inflammation, lungs, neutrophils, occupational asthma

## Abstract

The aryl hydrocarbon receptor (AhR) is a ligand-activated transcription factor that regulates the metabolism of xenobiotics. There is growing evidence that the AhR is implicated in physiological processes such proliferation, differentiation, and immune responses. Recently, a role of the AhR in regulating allergic asthma has been suggested, but whether the AhR also regulates other type of asthma, particularly occupational/irritant-induced asthma, remains unknown. Using AhR-deficient (*Ahr^−/−^*) mice, we compared the function of the AhR in the response to ovalbumin (OVA; allergic asthma) vs. chlorine (Cl_2_; irritant-induced asthma) exposure. Lung inflammation and airway hyperresponsiveness were assessed 24h after exposure to Cl_2_ or OVA challenge in *Ahr^−/−^* and heterozygous (*Ahr^+/−^*) mice. After OVA challenge, absence of AhR was associated with significantly enhanced eosinophilia and lymphocyte influx into the airways of *Ahr^−/−^* mice. There were also increased levels of interleukin-4 (IL-4) and IL-5 in the airways. However, OVA-induced airway hyperresponsiveness was not affected. In the irritant-induced asthma model caused by exposure to Cl_2_, the AhR did not regulate the inflammatory response. However, absence of AhR reduced Cl_2_-induced airway hyperresponsiveness. Collectively, these results support a differential role for the AhR in regulating asthma outcomes in response to diverse etiological agents.

## Introduction

Exposure of the lungs to environmental toxicants such as pesticides, solvents, and air pollutants may lead to acute and chronic pulmonary inflammation that is associated with the development of asthma (Wong et al., 2016). With the increased prevalence in the second half of the 20th century, it is estimated that over 300 million people world-wide have asthma (Braman, 2006; Asher et al., 2020), making it a major health burden (Boonpiyathad et al., 2019). Asthma is a heterogeneous chronic disease of the airways characterized by inflammation, airway hyperresponsiveness and narrowing that result from airway smooth muscle (ASM) contraction and airway remodeling (James et al., 2009; Asher et al., 2020). In susceptible individuals, these pathological characteristics cause recurrent episodes of wheezing, breathlessness, chest tightness, and coughing (Maslan and Mims, 2014).

Asthma is classified as allergic asthma or non-allergic asthma, and this is based on the age of onset (e.g., adult-onset asthma or childhood asthma). Also it can clinically be classified by the patient history, symptoms and the predominant type of leukocytes in the sputum (e.g., eosinophilic, neutrophilic, or paucigranulocytic; Simpson et al., 2006; Barnes, 2018; Papi et al., 2018). Eosinophilic asthma is one of the most common subtypes of asthma diagnosed in children and adults and can be either allergic or non-allergic. The allergic form is an adaptive T helper 2-driven disease characterized by elevated levels of interleukin (IL)-5, interleukin-4 (IL-4), and IL-13, associated with enhanced levels of circulating and lung eosinophils, elevated serum IgE, mucus hypersecretion and airway hyperresponsiveness (Holgate and Polosa, 2008; Chiba et al., 2009; Pelaia et al., 2015). In the non-allergic form, innate lymphoid cells (ILC2) produce IL-5 to recruit eosinophils into the airway (Jonckheere et al., 2019). Neutrophilic-asthma (non-eosinophilic) is triggered by Th1 and Th17 lymphocytes with the release of cytokines (e.g., IFN-γ and IL-17) which favor the development of a cellular immune response, activation of macrophages, and release of neutrophil chemokines (Papi et al., 2018). Environmental stimuli such as chlorine (Cl_2_) can trigger Th1 and Th17-mediated airway inflammation that is implicated in the development of severe neutrophilic asthma (Fisk et al., 2010; Pelaia et al., 2015). Chlorine and Cl_2_ derivatives are present in disinfecting agents that are widely used by cleaning personnel and are linked to the development of occupational asthma (De Genaro et al., 2018). Chronic low dose exposure to chlorine also occurs through frequentation of chlorinated swimming pools (Ferrari et al., 2011). Thus, exposure to environmental toxicants not only contributes to the increasing prevalence of asthma, but these exposures can also affect disease outcomes.

The molecular and cellular mechanisms involved in the pathogenesis of the asthmatic phenotype particularly allergic vs. non-allergic asthma are not fully understood. Recently, it has been shown that the aryl hydrocarbon receptor (AhR) may be involved in suppressing the development of allergic asthma (Jeong et al., 2012; Chang et al., 2020). The AhR is a ligand-activated transcription factor that belongs to the basic helix loop helix (bHLH)/PER-ARNT-SIM (PAS) family and is highly expressed in the lung. Historically the AhR is known for its ability to mediate the deleterious effects of the environmental toxicant 2,3,7,8-tetrachlorodibenzo-p-dioxin (TCDD; dioxin). In the absence of ligand, the AhR remains in the cytoplasm. After ligand binding, it translocates to the nucleus and forms a heterodimer with the AhR nuclear transporter (ARNT). This complex binds to DNA sequences termed the dioxin response element (DRE), initiating the transcription of genes that comprise the AhR gene battery such as cytochrome P450 (CYP) enzymes (Guerrina et al., 2018). Although historically, the AhR has been largely associated with xenobiotic metabolism leading to toxicity, we have shown that the AhR suppresses the development of chronic obstructive pulmonary disease (COPD; Guerrina et al., 2021), an obstructive lung disease caused predominantly by cigarette smoke. Mechanistically, the AhR also suppresses neutrophil recruitment to the lungs in response to cigarette smoke (Thatcher et al., 2007; De Souza et al., 2014; Rico De Souza et al., 2021). While a role of the AhR in controlling asthma related-outcomes has emerged (Xu et al., 2015; Thatcher et al., 2016; Chang et al., 2020; Poulain-Godefroy et al., 2020), these studies utilized mouse models of eosinophilic allergic asthma. However, a role for the AhR in suppressing asthma caused by other environmental triggers, particularly those that are associated with neutrophilic asthma, remains unknown.

Therefore, we sought to understand whether the AhR can control the development of the asthmatic phenotype using two different triggers: ovalbumin (OVA) and Cl_2_. Ovalbumin induces an eosinophilic asthma phenotype and thus is a model of allergen-induced asthma. For the second model, we used acute Cl_2_ exposure as a model of neutrophilic asthma. In these two models, we set out to study the extent of airway and parenchymal inflammation as well as airway hyperresponsiveness using AhR knock out (*Ahr^−/−^*) mice. Herein, we demonstrated an important role of the AhR in decreasing pulmonary inflammation in the OVA mouse model, but not in the Cl_2_ mouse model. These data highlight the differential role that AhR may play in controlling asthma phenotypes.

## Materials and Methods

### Chemicals

All chemicals were purchased from Sigma (St. Louis, MO, United States) unless otherwise indicated. 6-Formylindoleo [3,2-b] carbazole (FICZ) was from Tocris Bioscience (Minneapolis, MN, United States).

### Mice

Mice heterozygotes for AhR (*Ahr^+/−^*) and knockout (*Ahr^−/−^*) mice (strain B6.129-Ahr^tm1Bra^) were bred and maintained in the Research Institute of the McGill University Health Centre (RI MUHC) as previously described (Rico De Souza et al., 2021). This strain carries a targeted deletion of exon 2 of the *Ahr* gene and was backcrossed for 12 generations onto C57BL/6. As *Ahr^+/+^* or *Ahr^+/−^* mice do not exhibit any phenotypic difference in the ability to be activated by AhR ligands (Thatcher et al., 2007; De Souza et al., 2014). The *Ahr^+/−^* mice were used as littermate controls in this study. Mice were maintained on an *ad libitum* diet with free access to food and water and subjected to a 12-h light cycle. Male and female mice were used in experiments unless otherwise indicated. All animal procedures were approved by the McGill University Animal Care Committee (2,010–5,933), were carried out in accordance with the guidelines of the Canadian Council on Animal Care and followed the ARRIVE guidelines for the design, analysis, and reporting of research with animals (Kilkenny et al., 2010).

### Cl_2_ Exposure

Chlorine exposure was performed as previously described (Allard et al., 2019). Briefly, 8–12-week-old mice were exposed to Cl_2_ for 5min using a nose-only exposure device at a concentration of 100ppm. Chlorine was mixed with room air using a standardized calibrator (VICIMetronics, Dynacalibrator, Model230-28A). The AhR ligand FICZ was dissolved in DMSO and administered intraperitoneally (i.p.; 1μg per mouse). A single injection of FICZ or DMSO was given to mice on day 0 and 1h before the Cl_2_ exposure. Mice were sacrificed 24h after the Cl_2_ exposure.

### Ovalbumin Exposure

Mice were sensitized by i.p injection of 1mg/ml chicken OVA solution mixed with Imject alum adjuvant (1:4 dilution in PBS; Thermofisher Scientific). The control group received 200μl PBS mixed with of Imject alum alone. After 14days, sensitized mice were challenged on days 14, 15, and 16 by intranasal administration of 10μg of OVA diluted in 30μl of PBS. The PBS group received 30μl of PBS. Animals were sacrificed on day 18.

### Measurement of Airway Responsiveness

Mice were sedated with an i.p injection of xylazine (8mg/kg) and anaesthetized with i.p. injection of sodium pentobarbital (30mg/kg). Next, the mouse was tracheostomized using at 18-gauge cannula and connected to the flexiVent. Muscle paralysis was induced with rocuronium pentabromide (2mg/kg). Finally, the mouse was mechanically ventilated using the following settings [tidal volume of 10ml/kg, maximum inflation pressure of 30cm H_2_0, a positive end expiratory pressure (PEEP) of 3cm H_2_0 and a frequency of 150/min]. Following a standardized deep inflation, two lung inflations to a transrespiratory pressure of 25cm H_2_O were performed and baseline measurements were recorded in six replicates. Respiratory mechanics were estimated using a single compartment model and commercial software (Scireq). These included a low-frequency range of oscillations (1–20.5Hz) used to calculate Newtonian resistance (resistance_n_), an estimate of central airways resistance, and a single sinusoidal waveform (2.5Hz) used to calculate total respiratory system elastance and resistance. These procedures were also performed directly after inhalation of increasing concentrations of aerosolized methacholine (5–50mg/ml; Ano et al., 2017).

### Bronchoalveolar Lavage

Lungs were excised and PBS (0.5ml) was injected twice to lavage the lungs. The bronchoalveolar lavage (BAL) was centrifuged at 3,000rpm for 5min and the supernatant was separated from the cells. The cells were resuspended in PBS, counted and cytospin slides (CytoSpin, Thermofisher Scientific) were stained with HEMA 3 STAT PACK (Fisher Scientific).

### Analysis of Cytokines

Interleukin-4, IL-5, and IL-13 were quantified in BAL fluid collected as described above using a cytokine multiplex analysis (Milliplex MAP, Millipore) according to the manufacturer’s instructions and were read on a Luminex 100 System.

### Flow Cytometry

Lungs were collected, minced, and digested using collagenase IV at a concentration of 150units per ml at 37° C for 1h in RPMI medium containing 10% FBS. Single cell populations were then obtained by gently rubbing lung tissue over nylon mesh with 70μm pores. The nylon mesh was washed twice with medium and the lung homogenates were centrifuged at 1,500rpm for 5min at 4° C. Red blood cells were lysed by adding 2ml of ACK lysing buffer (Thermofisher Scientific) for 2min at room temperature. Cells were resuspended in 0.5ml of medium. Bronchoalveolar lavage cells were centrifuged and re-suspended. Lysis of red blood cells in BAL fluid was performed, when necessary, using ACK lysing buffer. Concentrations of all live cell suspensions were determined by trypan blue exclusion. Aliquots of 10^5^ for the BAL cells or 10^6^ for the lung cells were seeded in round-bottom 96-well plates. Fc receptors were then blocked with FC block anti-CD16/32 antibody (BD Biosciences) for 20min at 4°C. Cells were then labeled with viability dye (eFluor 780), anti-CD45 (Indo-1 violet) anti-Ly6G (Alexa fluor 700), anti-CD11c (APC), anti-SiglecF (PE), anti-CD3 (FITC), anti-CD4 (Pacific blue), and CD8 (PerCP.Cy5.5; BD Biosciences). Cells were analyzed immediately by flow cytometry. Cells were acquired using a BD FACSCanto flow cytometer (BD Biosciences) and data were analyzed by Flowjo software. Fluorescence minus one (FMO) was used to set the gates for the flow cytometry. Eosinophils were determined by first excluding the neutrophils (Ly6G^hi^). Eosinophils were identified as Ly6G^low^ CD11c^−/low^ Siglec-F^med/high^ as previously described (Abdala Valencia et al., 2016).

### Statistical Analysis

Results are reported as means±SEM. Statistical differences between group-mean values were determined using Prism 6 (GraphPad software) by two-way ANOVA followed by the Tukey’s multiple comparisons test. A value of *p*<0.05 was considered statistically significant.

## Results

### The AhR Decreases Airway Inflammation in OVA-Induced Allergic Asthma

**Figure 1:**
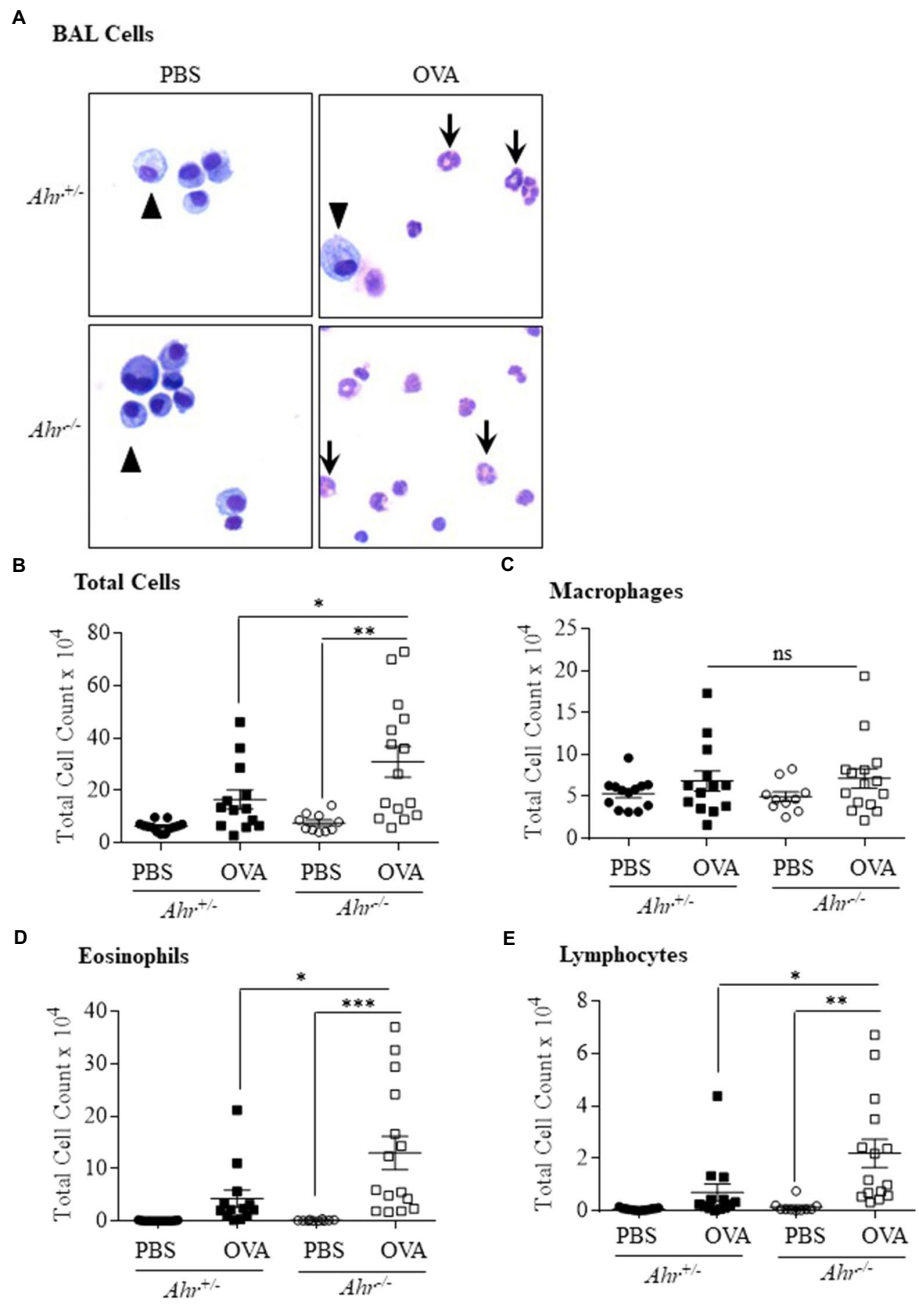
Aryl hydrocarbon receptor (AhR) reduces ovalbumin (OVA)-induced airway inflammation. **(A)** Bronchoalveolar lavage (BAL) cells – presence of macrophages (arrowheads) in the BAL as the predominant cell type in PBS-exposed mice. There were more eosinophils (arrows) in the OVA-exposed *Ahr^−/−^* as well as *Ahr^+/−^* mice. **(B)** Total Cells – there was a significant increase in total cells in *Ahr^−/−^* mice exposed to OVA (^**^p =0.001 OVA compared with PBS; ^*^p =0.0451 OVA-exposed *Ahr^−/−^* mice vs. OVA-exposed *Ahr^+/−^* mice). **(C)** Macrophages – there were no significant differences in macrophages numbers between the *Ahr^−/−^* and *Ahr^+/−^* exposed to OVA. **(D)** Eosinophils – there was a significant increase in eosinophils in OVA-exposed *Ahr^−/−^* mice compared with both PBS control (^***^p =0.0005) as well as OVA-exposed *Ahr^+/−^* mice (^*^p =0.0148). **(E)** Lymphocytes – the number of lymphocytes in OVA-exposed *Ahr^−/−^* mice was significantly higher than in OVA-exposed *Ahr^+/−^* mice compared with PBS control mice (^**^p =0.0016) as well as OVA-exposed *Ahr^+/−^* mice (^*^p =0.018). Results are expressed as the mean±SEM; values for individual mice from two independent experiments are shown.

**Figure 2:**
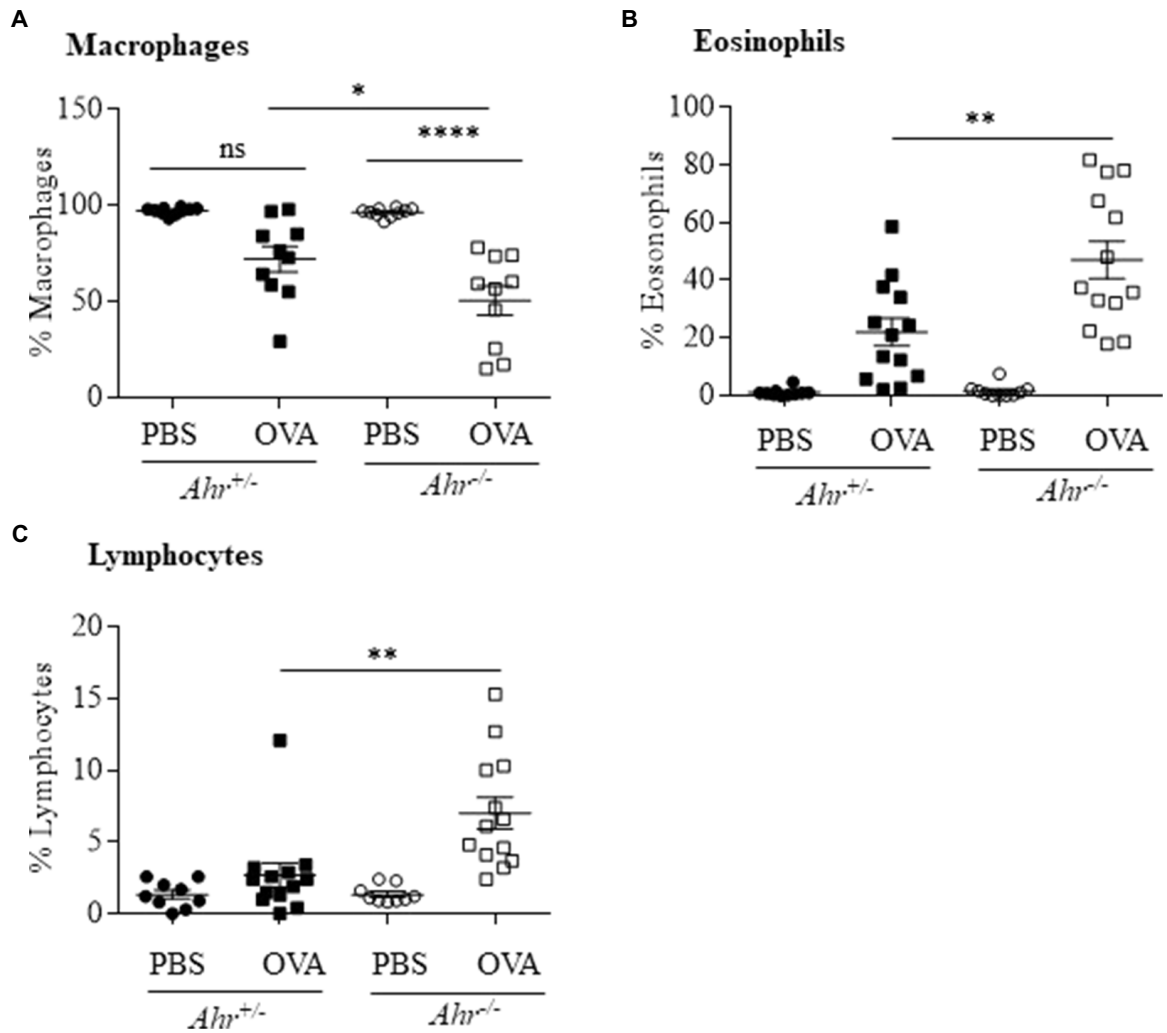
Percentage of immune cells in OVA-induced airway inflammation. **(A)** Macrophages – there was a significant difference in the percentage of macrophages between OVA-challenged *Ahr^−/−^* and *Ahr^+/−^* mice (^*^p =0.0232; ^****^p =0.0001 between PBS and OVA-challenged *Ahr^−/−^* mice). **(B)** Eosinophils – there was a significantly higher percentage of eosinophils in OVA-exposed *Ahr^−/−^* mice compared with OVA-exposed *Ahr^+/−^* mice (^**^p =0.0011). **(C)** Lymphocytes – there was a significantly higher percentage of lymphocytes in OVA-exposed *Ahr^−/−^* mice compared with OVA-exposed *Ahr^+/−^* mice (^**^p =0.0016). Results are expressed as the mean±SEM from two independent experiments.

As OVA is a well-characterized allergic asthma model (Gueders et al., 2009; Pareek et al., 2019), we utilized this model to test the importance of AhR expression on the suppression of this asthma phenotype. Here, evaluation of BAL cells in mice sensitized and challenged with OVA revealed that OVA significantly increased the number of total cells compared with the PBS group in both *Ahr^−/−^* and *Ahr^+/−^* mice (Figures 1A,B). Total BAL cells were significantly higher in OVA-exposed *Ahr^−/−^* mice compared with OVA-exposed *Ahr^+/−^* mice (Figure 1B). While there was no difference in macrophage numbers (Figure 1C), there were significantly more eosinophils and lymphocytes in the airways of mice sensitized and challenged with OVA in both *Ahr^−/−^* and *Ahr^+/−^* mice compared with PBS control mice (Figures 1D,E). Reflecting the increase in total cell numbers in *Ahr^−/−^* mice, there were also significantly more eosinophils and lymphocytes in the *Ahr^−/−^* mice compared with *Ahr^+/−^* mice; the percentages of eosinophils and lymphocytes were also significantly higher (Figure 2). Neutrophils were not detected. Thus, these data recapitulate that the AhR suppresses eosinophilic airway inflammation in an allergic model.

### The AhR Reduces Activated Eosinophils in Lung Tissue During OVA-Induced Allergic Asthma

**Figure 3:**
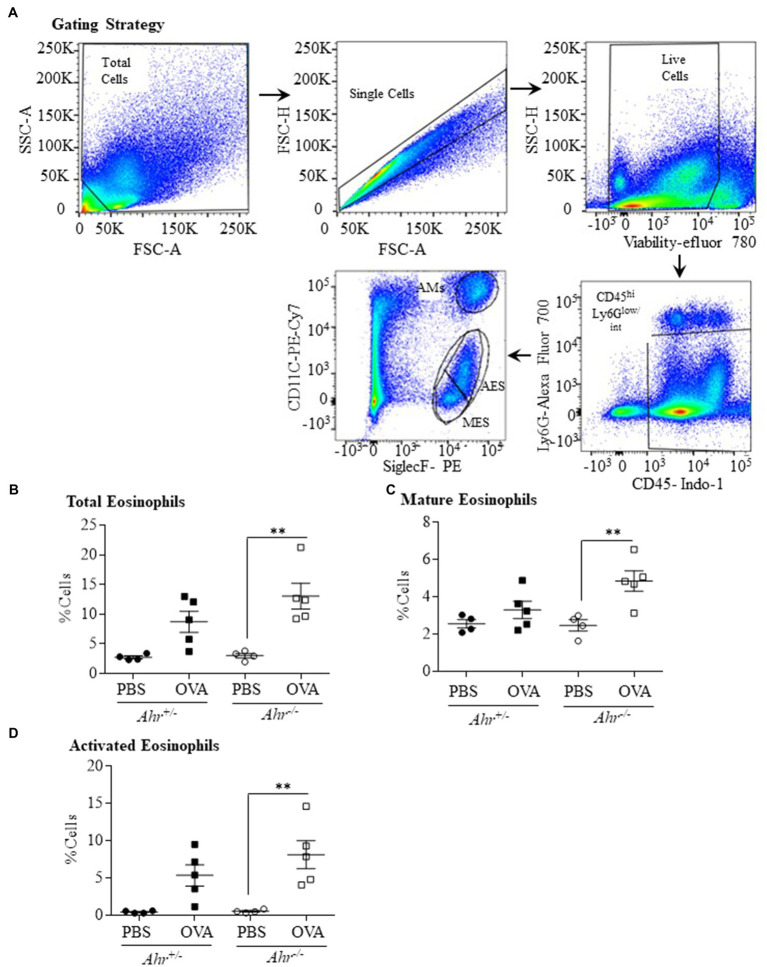
Aryl hydrocarbon receptor expression decreases the percentage of eosinophils in the lung parenchyma after exposure to OVA. **(A)** Gating Strategy – the gating strategy utilized for flow cytometry to quantify mature vs. activated eosinophils in lungs tissue is shown. The percentage of total **(B)**, mature **(C)**, and activated **(D)** eosinophils in lung tissue was significantly increased in *Ahr^−/−^* mice exposed to OVA compared with PBS control mice (^**^p =0.0028; 0.0088, and 0.0065, respectively). Results are expressed as the mean±SEM; values for individual mice are shown.

Our finding that the AhR reduces allergen-induced eosinophil influx into the airways led us to speculate whether this suppression also occurred in the lung parenchyma. To more comprehensively profile the eosinophil phenotype, lung cells from OVA-challenged mice were isolated 48h post challenge, and mature (SiglecF^int^ CD11c^−^) and activated (SiglecF^hi^ CD11c^lo^) eosinophils were identified by flow cytometry. The gating strategy used to quantify mature vs. activated eosinophils is presented in Figure 3A (Abdala Valencia et al., 2016). There was a significant increase in total eosinophils only in the lung tissue of the OVA-exposed *Ahr^−/−^* mice compared with PBS controls (Figure 3B) but no change in total eosinophils was found in OVA-exposed *Ahr^+/−^* mice. There was also a significant increase in both mature (Figure 3C) and activated (Figure 3D) eosinophils in OVA-exposed *Ahr^−/−^* mice compared with PBS-exposed *Ahr^−/−^* mice. Overall, these new data suggest that *Ahr^−/−^* mice challenged with OVA recruit more eosinophils into the lung, which subsequently upregulate CD11c, after which they migrate into the airways. This enhanced response does not occur in *Ahr^+/−^* mice.

### The *Ahr ^−/−^* Mice Have Increased IL-4 and IL-5 in the BAL

**Figure 4:**
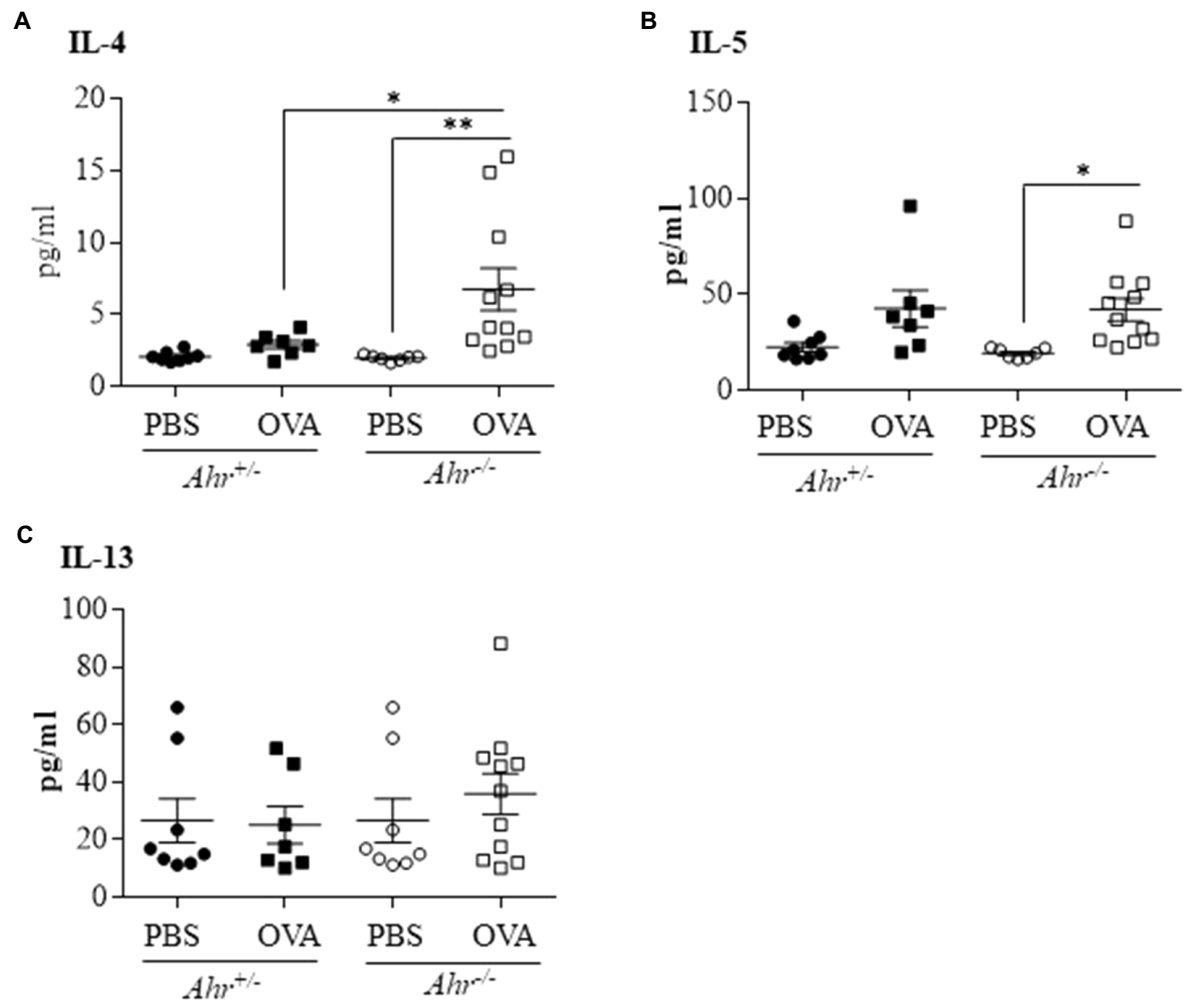
*Ahr^−/−^* mice exhibit increased levels of IL-4 and IL-5 following OVA challenge. Protein levels of interleukin-4 (IL-4), IL-5, and IL-13 were measured in the BAL fluid by multiplex assay. **(A)** IL-4 – there was a significant increase of IL-4 in the *Ahr^−/−^* mice exposed to OVA compared with the *Ahr^−/−^* mice exposed to PBS (^**^p =0.0095) and to the *Ahr^+/−^* mice exposed to OVA (^*^p =0.0466). **(B)** IL-5 – there was a significant increase of IL-5 only in the *Ahr^−/−^* mice exposed to OVA (^*^p =0.0396). **(C)** IL-13 – IL-13 was not significantly increased between any of the groups. Results are expressed as the mean±SEM; values for individual mice are shown.

Because, we observed that the AhR reduces eosinophil recruitment into the lungs, we sought to determine whether the AhR regulates the secretion of these Th2 cytokines in OVA-challenged mice. Using a multiplex assay to quantify levels of IL-4, IL-5, IL-13 in the BAL fluid, we found that there was a significant increase in IL-4 (Figure 4A) and IL-5 (Figure 4B) only in *Ahr^−/−^* mice after OVA challenge. IL-4 was also significantly higher in OVA-exposed *Ahr^−/−^* mice compared with the *Ahr^+/−^* mice (Figure 4A). Interestingly, there was no significant change in IL-13 in any of the groups (Figure 4C).

### The AhR Does Not Affect Lung Function in the OVA-Induced Allergic Asthma Model

**Figure 5:**
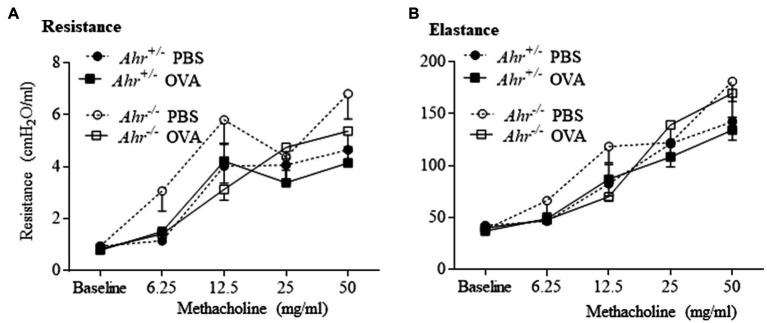
Aryl hydrocarbon receptor does not influence lung function in the allergic asthma model. Lung mechanics were evaluated by flexiVent. There was no significant difference between OVA-immunized *Ahr^−/−^* and *Ahr^+/−^* mice in any of the parameters evaluated including resistance **(A)** and elastance **(B)**. Results are expressed as the mean±SEM.

Next, we investigated whether the AhR regulates airway hyperresponsiveness in OVA challenged mice using a flexiVent to measure airway resistance upon exposure with increasing concentrations of aerosolized methacholine. Consistent with the lack of change in levels of IL-13, there was no significant difference in resistance and elastance between OVA-exposed *Ahr^−/−^* and *Ahr^+/−^* mice (Figures 5A,B). Thus, although the AhR controls immune cell infiltration to the lungs in the OVA asthma model, the AhR exerts minimal influence on airway function.

### Irritant-Induced Inflammation Is Independent of the AhR

**Figure 6:**
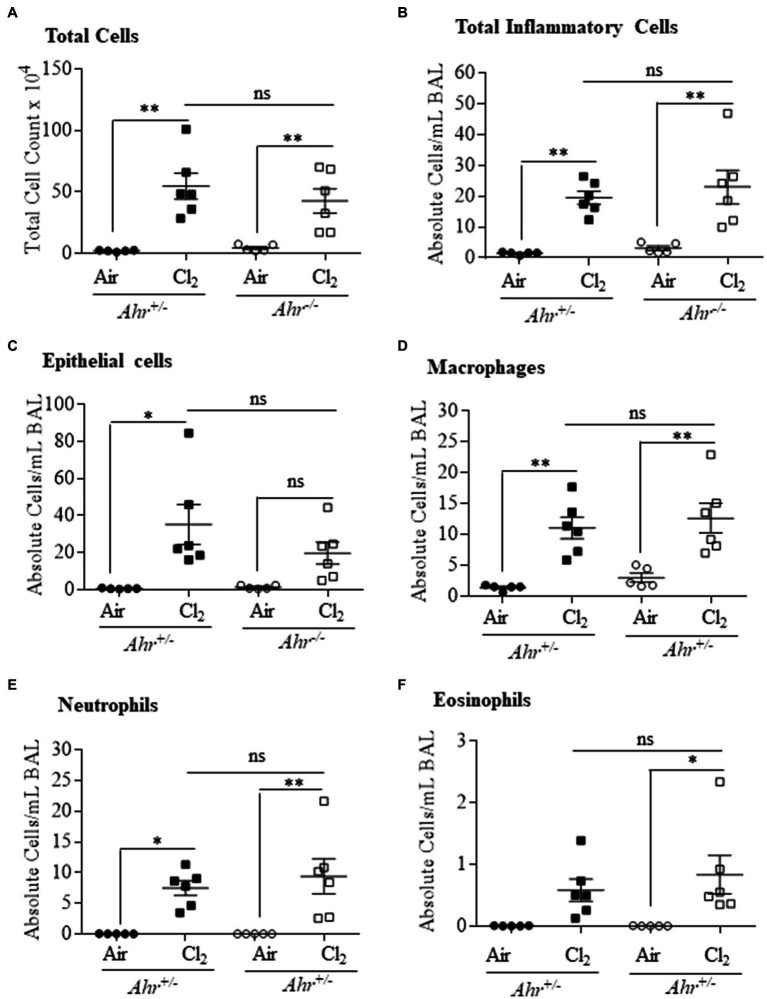
Aryl hydrocarbon receptor does not control chlorine (Cl_2_)-induced airway inflammation. Total and differential cell counts of the BAL of *Ahr^+/−^* and *Ahr^−/−^* was evaluated 24h after exposure to Cl_2._ There was a significant increase in total cells **(A)**, total inflammatory cells (^*^p =0.0023; **B**), epithelial cells (^*^p =0.06001; **C**), macrophages ^**^p =0.0039 **(D)** and neutrophils (^*^p =0.0297; ^**^p =0.0055; **E**) and eosinophils (^*^p =0.0402; **F**) in mice exposed to Cl_2_ compared with the control mice. There was no significant difference (ns) between the *Ahr^+/−^* and *Ahr^−/−^* mice. Results are expressed as the mean±SEM; values for individual female mice are shown.

We next utilized a model of irritant-induced asthma that provokes a neutrophilic response in the lungs and airways to evaluate whether the AhR can also suppress neutrophilia in response to diverse etiologic agents. For these experiments, we utilized Cl_2_ as a representative trigger of the irritant-induced asthma phenotype. Here, airway inflammation was observed in both *Ahr^+/−^* and *Ahr^−/−^* mice after Cl_2_ exposure, where there was a significant increase in the number of total cells in the BAL in *Ahr^+/−^* and *Ahr^−/−^* mice exposed to Cl_2_ compared with air-only controls (Figure 6A). In addition, the level of inflammatory cell infiltration was also significantly increased in mice exposed to Cl_2_ (Figure 6B). Chlorine also caused a significant increase in the number of epithelial cells in the BAL of *Ahr^+/−^* mice; there was a trend toward an increase in *Ahr^−/−^* mice although this did not reach statistical significance (Figure 6C). Recruitment of inflammatory cells to the lungs in response to Cl_2_ was also significantly increased compared with air-exposed mice and was dominated by macrophages (Figure 6D) and neutrophils (Figure 6E). There was also a significant increase in the number of eosinophils with Cl_2_ exposure only in *Ahr^−/−^* mice (Figure 6F). However, there was no significant difference in any of these cell types between Cl_2_-exposed *Ahr^+/−^* and *Ahr^−/−^* mice.

### The AhR Regulates Airway Hyperresponsiveness in Response to Cl_2_-Induced Lung Damage

**Figure 7:**
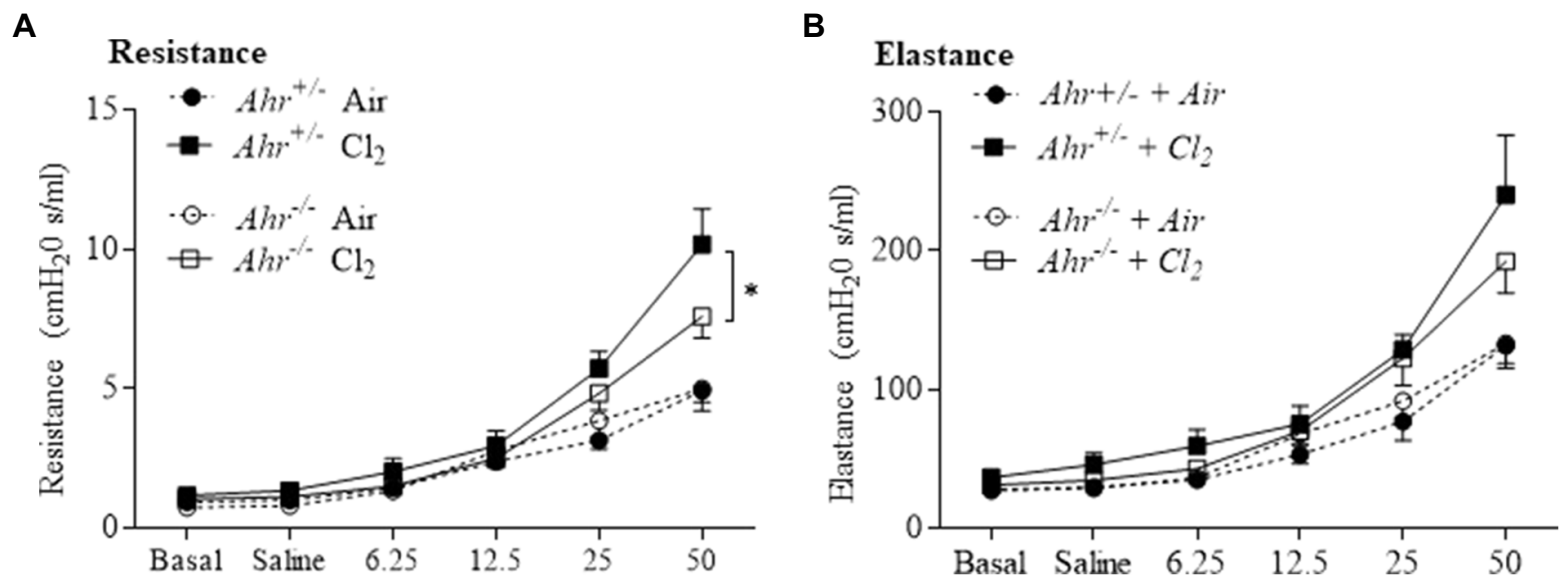
Aryl hydrocarbon receptor increases airway hyperresponsiveness after Cl_2_ exposure. Lung mechanics parameters included assessment of resistance **(A)** and elastance **(B)** by flexiVent in response to methacholine in *Ahr^+/−^* and *Ahr^−/−^* at 24h after exposure to air or Cl_2_. Cl_2_ increased resistance compared with the control. Respiratory resistance was significantly lower in *Ahr^−/−^* mice compared with *Ahr^+/−^* mice at the highest dose of methacholine (^*^p =0.0358). Results are expressed as the mean±SEM.

Next, we evaluated airway hyperresponsiveness after exposure to Cl_2_. These data revealed the general regulation of lung function by the AhR in this model. First, respiratory resistance, which reflects airway hyperresponsiveness to inhaled aerosolized methacholine, was greater in Cl_2_ exposed mice (Figure 7A). In the absence of AhR, resistance was significantly lower compared with *Ahr^+/−^* mice (Figure 7A). We also evaluated other parameters of respiratory mechanics such as elastance (Figure 7B). These data suggest that the AhR actually promotes airway hyperresponsiveness after Cl_2_ exposure.

### The Endogenous AhR Ligand FICZ Does Not Affect Cl_2_-Induced Lung Inflammation

**Figure 8:**
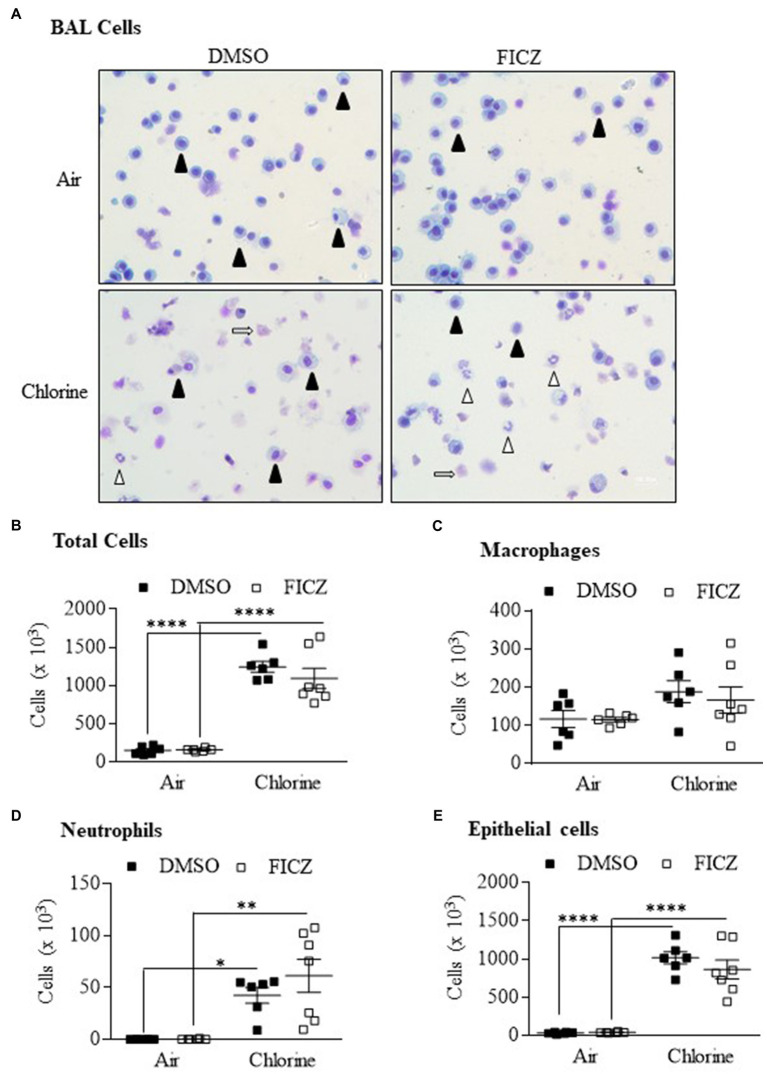
6-Formylindoleo [3,2-b] carbazole (FICZ) does not attenuate Cl_2_-induced airway inflammation. **(A)** BAL cells – there was an increase in neutrophils (open arrowheads) and epithelial cells (open arrows) 24h after exposure to Cl_2_. Macrophages are indicated as closed arrowheads. **(B)** Total Cells – there was a significant increase in total cells in mice exposed to Cl_2_ (^****^p =0.0001). FICZ had no effect on the total number of cells. **(C)** Macrophages – FICZ did not change macrophages in response to Cl_2_. **(D)** Neutrophils – there was a significant increase in neutrophils in response to Cl_2_ (^*^p =0.0313 and ^**^p =0.001 in DMSO and FICZ treated mice, respectively). **(E)** Epithelial cells – there was a significant increase in BAL epithelial cells in mice treated with DMSO or FICZ and exposed to Cl_2_ (^****^p =0.0001). There was no significant difference between FICZ and DMSO-treated mice exposed to Cl_2_. Results are expressed as the mean±SEM; values for individual male mice are shown.

6-Formylindoleo [3,2-b] carbazole is an endogenous AhR ligand and derivative of tryptophan that is produced in the skin after ultraviolet exposure (Wei et al., 1998; Wincent et al., 2009). Our data show that FICZ attenuates acute lung neutrophilia caused by cigarette smoke (Rico De Souza et al., 2021). Therefore, we next tested whether activation of the AhR by FICZ would protect against lung inflammation in response to Cl_2_. Using *Ahr^+/−^* mice, these data show that there was a significant increase in the total BAL cells from Cl_2_ exposure (Figures 8A,B). There was no increase in macrophages (Figure 8C). There was a significant increase in both neutrophils (Figure 8D) and epithelial cells (Figure 8E) in response to Cl_2_. However, FICZ did not significantly change the levels of cells in the BAL in response to Cl_2_, suggesting that FICZ does not alter the inflammatory response to Cl_2_.

## Discussion

Asthma is a complex disease triggered by environmental factors that lead to dysregulation of the immune system. Herein, we report the novel findings that there is differential regulation of the asthma phenotype between OVA and Cl_2_. Our novel results show that expression of the AhR had minimal effect on Cl_2_-induced neutrophilia, but attenuated OVA-induced pulmonary inflammation that is typified by eosinophilia. This latter finding is consistent with previous publications showing that AhR-deficient mice developed allergic asthma in preclinical OVA and cockroach allergen models (Xu et al., 2015; Thatcher et al., 2016; Chang et al., 2020). These confirmatory data strongly support the general regulation of the allergic asthma phenotype by the AhR. However, one of the unresolved questions in AhR biology is why there can be similar responses in ligand-treated mice and AhR deficient mice. In the context of asthma, AhR activation by TCDD or benzo[*a*]pyrene (B[*a*]P) can be pro-inflammatory and induce *MUC5AC* expression, leading to mucus hypersecretion, airway remodeling, dysregulation of antigen-presenting cells, and exacerbation of asthma (Wong et al., 2018; Poulain-Godefroy et al., 2020). Yet, our data herein confirm that AhR expression also protects against the development of allergic asthma. Although, we did not measure AhR levels, we posit that this discrepancy may be related to changes in AhR expression after its activation. Because a key event in AhR-mediated signaling is prolonged AhR degradation following ligand binding (Prokipcak and Okey, 1991; Pollenz, 2007), it could be that decreased AhR expression due to persistent activation by TCDD or B[*a*]P is sufficient to mimic an *Ahr^−/−^* phenotype, thereby yielding similar results. This is supported by the observation that both *Ahr^−/−^* mice and TCDD-exposed mice have impaired hippocampal neurogenesis (Latchney et al., 2013). This may also be why both *Ahr^−/−^* cells as well as cells exposed to AhR ligands exhibit decreased proliferation (Elizondo et al., 2000; Faust et al., 2013; Hecht et al., 2014). Thus, overall AhR expression levels in the lungs may be an important determinant of its ability to mitigate damage associated with environmental exposures, and further highlight the importance of the AhR in the maintenance of lung health in response to diverse environmental exposures (Guerrina et al., 2018).

As previous work has shown that *Ahr^−/−^* mice have enhanced airway inflammation and hyperresponsiveness in response to OVA (Chang et al., 2020), our results are comparable to previous publications in the allergic asthma models in that there is suppression of airway inflammation by the AhR, including reduced infiltration of eosinophils and lymphocytes (Chang et al., 2020). There are, however, differences in the response between these studies and ours. For example, Chang et al. (2020) reported that there was an increase in macrophages and neutrophils in the OVA model which was not observed in our study. We also did not see an increase in IL-13 as reported by others (Chang et al., 2020). Finally, our results also differ from that of Chang et al. (2020) in that *Ahr^−/−^* mice in their study exhibited an enhancement of airway hyperresponsiveness after OVA immunization. These variations in results could be due to differences in the protocol in OVA administration and/or duration between studies. However, despite these differences, our results further reaffirm the importance of the AhR in suppressing inflammation associated with the allergic asthma phenotype.

Although, the AhR mitigates allergic asthma, whether the AhR affects the pathogenesis of other types of asthma, such as irritant-induced asthma, was not known. Surprisingly, the AhR did not regulate inflammation, including neutrophilia in response to Cl_2_. Based on the lack of difference in immune cell infiltration due to AhR expression, we did not measure cytokine production in this model. However, Cl_2_ is a gas that causes oxidative stress and airway dysfunction following inhalation, the effects of which can be ameliorated by administration of antioxidants (Ano et al., 2017). It is known that AhR-deficient mice are more susceptible to hyperoxic lung injury due to decreased expression of antioxidant enzymes such as cytochrome P4501A, NAD(P)H quinone reductase-1 (NQO1) and microsomal glutathione S-transferase (GST; Zhang et al., 2015). Despite not having an effect on the inflammatory response, the AhR did aggravate Cl_2_-induced airway hyperresponsiveness. Persistent airway hyperresponsiveness is caused by inflammatory and structural changes in the airways (Gabehart et al., 2013). Our data suggest that changes in the inflammatory response are unlikely to be how the AhR aggravates the airway reactivity after Cl_2_ exposure. Other possibilities to explain these results include that the AhR may have direct effects in tissues that influence airway hyperresponsiveness such as the epithelium or smooth muscle.

As Th2-mediated responses that underlie airway eosinophilia and airway hyperresponsiveness have been linked to IL-4, IL-5, and IL-13, we measured the levels of these cytokines in the BAL of OVA-exposed mice. Of these, IL-4 and IL-5 were significantly increased in *Ahr^−/−^* mice, a finding that may explain the increased lung eosinophil influx (Kips et al., 2001; Maes et al., 2012). These results also supported an important role for the AhR in suppressing lung inflammation and are consistent with previous studies demonstrating an anti-inflammatory role for the AhR in asthma models, including studies that also utilized cockroach allergen challenge (Xu et al., 2015; Thatcher et al., 2016). Our results are therefore consistent with the hypothesis that the AhR serves as an important negative regulator of inflammation in the lungs. However, one of the limitations of our study is the focus on an acute model of chlorine and OVA exposure, as this does not allow for the development of structural changes and airway remodeling, pathological features of asthma that contribute to the clinical manifestations of the disease. Other limitations of the OVA model include that the pattern and distribution of lung inflammation in the lower airway of mice differs from humans due to differences in lung branching (Kim et al., 2019). It also needs to be noted that there are differences between these asthma models in terms of the number and duration of the exposure (e.g., three nasal OVA challenges vs. a single chlorine) that may influence interpretation of these data. The adaptation of mice to repeated chlorine exposures prevents the application of identical exposure protocols (Allard et al., 2019). However, despite these limitations, these exposure regimes allowed us to compare the role of the AhR using two models of exposure that induce different asthma phenotypes.

Thus, we show that AhR differentially affects the development asthma-like disease, with the majority of AhR-dependent effects involving the suppression of inflammation associated with the allergic phenotype. In conjunction with our previous work establishing the AhR attenuates tobacco smoke-induced inflammation (Rogers et al., 2017; Rico De Souza et al., 2021), these findings position the AhR as a homeostatic modulator of pulmonary inflammation in response to diverse etiologic agents. A better understanding of the connection between the AhR and its role in pulmonary inflammation may aid the development of therapeutic agents to combat specific inflammatory lung diseases.

## Data Availability Statement

The raw data supporting the conclusions of this article will be made available by the authors, without undue reservation.

## Ethics Statement

All animal procedures were approved by the McGill University Animal Care Committee and were carried out in accordance with the Canadian Council on Animal Care (Protocol Number: 5933).

## Author Contributions

HT, MS, AR, and BA: data curation and/or analysis. CB: funding acquisition. HT, AR, BA, VM, and JM: methodology. HT and CB: project administration. CB and EF: supervision. HT, CB, DE, EF, VM, ZH, and JM: intellectual contributions. HT, ZH, CB, DE, JM, and EF: manuscript writing, review, and editing. All authors contributed to the article and approved the submitted version.

## Funding

This work was supported by the Canada Foundation for Innovation (CFI), the Canadian Institutes for Health Research Project Grants (168836 and 162273), and the Natural Sciences and Engineering Research Council of Canada (NSERC). CB was supported by a salary award from the Fonds de recherche du Quebec-Sante (FRQ-S). HT was supported by a Réseau de recherche en santé réspiratoire du Québec (RSR) Scholarship and a Meakins-Christie Laboratories Collaborative Research Award.

## Conflict of Interest

The authors declare that the research was conducted in the absence of any commercial or financial relationships that could be construed as a potential conflict of interest.

## Publisher’s Note

All claims expressed in this article are solely those of the authors and do not necessarily represent those of their affiliated organizations, or those of the publisher, the editors and the reviewers. Any product that may be evaluated in this article, or claim that may be made by its manufacturer, is not guaranteed or endorsed by the publisher.
